# Effects of Training Interventions on Physical Fitness and Performance in Kabaddi Players: A Systematic Review

**DOI:** 10.3390/sports14010037

**Published:** 2026-01-13

**Authors:** Daniel González-Devesa, Lidia Iglesias Vieites, Nerea Blanco-Martínez

**Affiliations:** 1 Grupo de Investigación en Actividad Física, Educación, y Salud (GIAFES), Universidad Católica de Ávila, C/Canteros, 05005 Ávila, Spain; 2 IES de Teis, Consellería de Educación, Ciencia, Universidades y Formación Profesional, Xunta de Galicia, 36216 Vigo, Spain; lidiai@edu.xunta.gal; 3 Departamento de Didácticas Especiáis, Universidade de Vigo, 36310 Vigo, Spain; n.blanco@uvigo.gal; 4 Well-Move Research Group, Galicia Sur Health Research Institute (IIS Galicia Sur), SERGAS-UVIGO, 36310 Vigo, Spain

**Keywords:** team sports, exercise, physical fitness, physical conditioning

## Abstract

Kabaddi is a contact sport that demands high physical fitness and specific technical skills. Although multiple training programs have been designed to improve performance, no systematic review had previously synthesized their effects on physical, physiological, and performance-related outcomes. This systematic review of randomized controlled trials aimed to evaluate the impact of different training interventions on physical fitness, physiological parameters, and sport performance in kabaddi players. A systematic review was performed in the Web of Science, PubMed, and Scopus databases up to May 2025. Randomized controlled trials analyzing training interventions in kabaddi players were included. Ten studies with a total of 458 participants were included. Interventions lasted between 6 and 12 weeks and comprised modalities such as strength training, plyometrics, combined training, circuit training, SAQ (speed, agility, and quickness), and Tabata. Nine studies reported significant within-group improvements in variables such as strength, speed, agility, and flexibility. In studies with between-group comparisons, training interventions could be more effective than control conditions. One study also reported improvements in physiological and hematological parameters. Overall, training interventions in kabaddi players may improve physical fitness, sport-specific performance, and certain physiological and hematological parameters. However, the evidence should be interpreted with caution given the predominantly fair methodological quality of the included trials. PROSPERO (CRD420251272758).

## 1. Introduction

The Kabaddi is a contact team sport originating from South Asia, specifically India, which has gained worldwide popularity in recent years [1,2]. Although it was historically played on grass, sand, or other soft surfaces, the modern version of Kabaddi is played indoors on a synthetic rubber mat that conforms to specific dimensions [3,4]. Since its inclusion in the Asian Games in 1990 and the launch of the Pro Kabaddi League (PKL) in 2014, the sport has experienced significant growth in both popularity and professionalization, establishing itself as a symbol of Asian sports [5,6].

This sport is characterized by its combative and high-intensity intermittent nature, combining elements of wrestling and agility in a constantly physical environment [7,8]. During the game, athletes alternate between offensive and defensive situations that demand both technical skills and high levels of physicality [1,2,9]. The primary objective is for an offensive player to invade the opposing team’s half, touch as many defenders as possible, and return to their own side without being caught [10]. This requires a precise combination of strength, speed, agility, and endurance, as well as strict breath control, since the attacker must continuously chant “kabaddi” during their raid to comply with the game’s unique rule [11].

Performance in kabaddi relies heavily on body composition, muscle function, and neuromuscular coordination, along with the ability to make quick decisions in high-stress, physically intense situations [7,11]. These factors are critical, as small differences in strength, speed, or agility can be the deciding factor between scoring a point and a successful counterattack by the opposing team [12,13,14,15]. For this reason, kabaddi training not only focuses on developing the technical skills of the game but also aims to optimize the physical parameters essential for competitive performance [16]. However, while regular kabaddi practice is known to significantly improve parameters such as strength, speed, agility, endurance, and flexibility [16,17,18], it remains uncertain whether this approach is more effective than other specialized training methods for developing these physical qualities.

Given this context and the potential benefits of optimized training for kabaddi players, this study aims to conduct a systematic review to compare the effects of different training methods on key physical fitness parameters such as strength, speed, flexibility, endurance, and agility specifically in kabaddi athletes. To the best of the authors’ knowledge, no comprehensive systematic reviews have been published to evaluate the advantages and limitations of various training approaches for this sport, providing evidence to enhance the physical preparation of both high-performance and recreational kabaddi practitioners. In light of this, the aim of this systematic review of randomized controlled trials is to compare the effects of various training methods on the physical fitness and sport performance of kabaddi players.

## 2. Methods

This systematic review and meta-analysis was carried out in accordance with the Preferred Reporting Items for Systematic Reviews and Meta-Analyses (PRISMA) guidelines [19] (Supplementary Material File S1). This systematic review was registered in PROSPERO (CRD420251272758).

### 2.1. Search Strategy

A systematic search was conducted across three electronic databases, Web of Science, PubMed, and Scopus, covering all records available up to May 2025. The search strategy employed only the term “kabaddi”, as the authors intended to perform a broad search to minimize the risk of excluding potentially relevant studies. In addition, a snowballing strategy was also used to identify further relevant studies.

### 2.2. Eligibility Criteria

Randomized controlled trials (RCTs) that reported results on the effects of training interventions in kabaddi players were eligible for inclusion in this review. Studies were excluded if they: (a) lacked a control or comparison group; (b) did not provide quantitative data; or (c) had no full-text available. No studies were excluded based on language. Book chapters, books, theses, dissertations, notes, and conference abstracts or communications were also excluded.

### 2.3. Study Selection

Two authors independently reviewed the titles and abstracts of the retrieved studies to determine their eligibility. Full-text versions of all potentially relevant studies were then obtained. When eligibility remained unclear, a third author was involved to help resolve disagreements and make a final decision. Furthermore, Google Scholar was consulted to find additional studies that met the inclusion criteria.

### 2.4. Data Extraction

For each included study, data were collected on the title, authors, year of publication, study design, participant characteristics, intervention details, measured outcomes, and main findings, as well as dropouts and adverse events. This information was systematically organized in Table 1. Data extraction was carried out by one researcher and subsequently verified by a second reviewer to ensure accuracy.

### 2.5. Quality Appraisal

The methodological quality of each RCT was obtained from the Physiotherapy Evidence Database (PEDro). When a study was not listed in PEDro, two authors independently assessed its quality, resolving any disagreements through consensus. The quality of studies was classified using the cut-off points suggested by Silverman et al. [30]: excellent (9–10), good (6–8), fair (4–5), and poor (≤3).

## 3. Results

**Figure 1:**
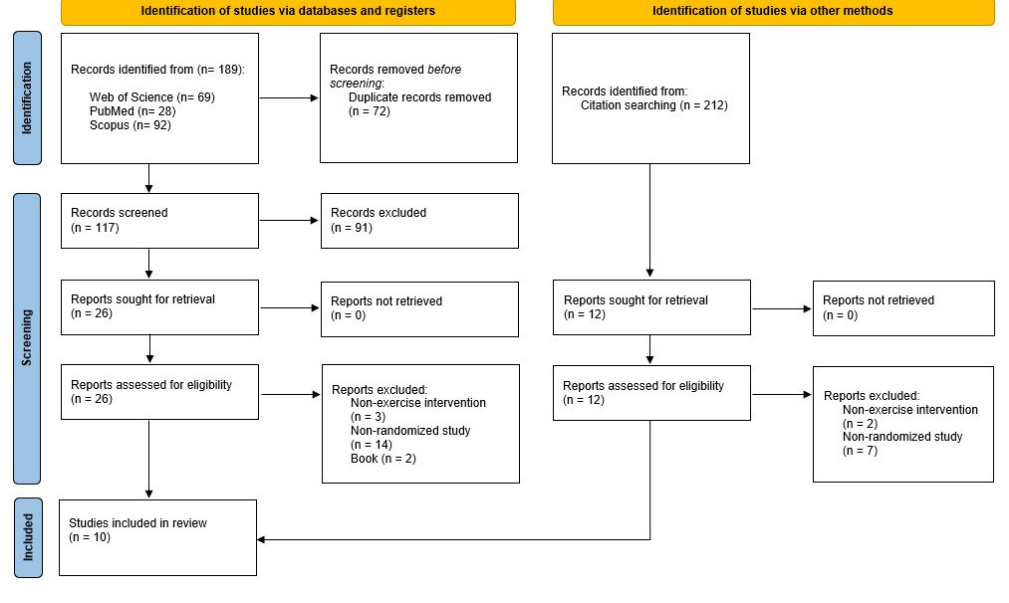
PRISMA (Preferred Reporting Items for Systematic Reviews and Meta-Analyses) study flow diagram.

A total of 189 records were identified through the database search. After removing duplicates, 117 titles and abstracts were screened, and subsequently, 26 articles were retrieved for the full-text assessment. Finally, 10 RCTs met the inclusion criteria and were included in the systematic review (Figure 1).

### 3.1. Study Characteristics

The reviewed studies were published between 2014 and 2024 and included a total of 458 participants. Of these, 353 were male and 105 were female. Only two studies included female participants in their samples [25,26], and none included mixed-gender samples. Sample sizes in individual studies ranged from 30 [21,23,27] to 80 [24] participants. Only one study involved elite players [22], eight studies included young adult players, and one study focused on school-aged adolescents [28]. Table 1 provides a summary of the main characteristics of the included studies.

### 3.2. Interventions Characteristics

The training interventions lasted between 6 and 12 weeks. Most studies reported a training frequency of 3 to 5 sessions per week, with the exception of studies Shanmugam et al. [24] and Karuppaiah & Kumar [23], which did not report session frequency. Session durations ranged from 60 to 90 min. However, four studies did not provide information regarding session duration. Only the study by Irandoust & Taheri [22] reported training intensity, specifying a range of 60–86% of 1 RM for the strength training group. No studies reported dropouts or adverse events during or following the interventions.

The training interventions included a variety of modalities such as strength training, plyometric training, combined training (strength and plyometric), circuit training, interval/continuous training, speed, agility and quickness (SAQ) training, game-specific training, and Tabata. Control groups maintained their usual lifestyle without engaging in any structured exercise programs.

### 3.3. Main Outcomes

#### 3.3.1. Physical Fitness

Nine of the ten studies included in this review assessed physical fitness parameters, including strength, speed, flexibility, and agility [20,21,22,24,25,26,27,28,29]. Four out of the five studies that examined muscular strength reported significant within-group improvements following the training interventions. Speed was assessed in five studies, and four of them reported within-group improvements in this variable. Flexibility was evaluated only in the study by Arjunan [20], which found significant within-group improvements in the intervention group. Agility was assessed in five studies, with four reporting significant within-group improvements in participants from the experimental groups. None of the included studies reported within-group improvements in the control groups for any of the physical fitness parameters analyzed.

In studies that included inter-group comparisons, the training programs were more effective than the control conditions in improving strength, speed, flexibility, and agility [20,21,22,25,26,27,28,29].

#### 3.3.2. Sports Performance Variables

Three studies investigated the effects of training interventions on sport-specific performance outcomes [21,23,26]. Nithin et al. [21] reported significant within-group improvements in the intervention group. Additionally, the experimental group outperformed the control group after the 12-week intervention. Sureshkumar et al. [26] found that the experimental groups demonstrated superior performance compared to the control group when evaluating kabaddi-specific skills after interventions. Karuppaiah & Kumar [23] demonstrated that 12 weeks of circuit training improved the specific skills assessed.

#### 3.3.3. Physiological Variables

Nithin et al. [21] analyzed physiological variables, including VO2_max_ and resting heart rate. Significant within-group improvements were observed in both variables after 12 weeks of Bulgarian Bag Training. Furthermore, the group that performed Bulgarian Bag Training showed superior outcomes compared to the control group.

#### 3.3.4. Hematological Variables

Nithin et al. [21] analyzed physiological variables including white blood cell count, red blood cell count, and hemoglobin levels. Significant within-group improvements were observed in all variables following the experimental intervention. Furthermore, the experimental group showed superior outcomes compared to the control group across all three variables assessed.

### 3.4. Methodological Quality

The methodological quality of the included RCT was considered poor in one study [23], fair in eight [20,22,24,25,26,27,28,29] and good in one [21] (Table 2).

## 4. Discussion

This systematic review gathered evidence from ten RCTs that evaluated the effects of various training methods on the physical fitness and sport performance of kabaddi players. Although the findings should be interpreted with caution due to the predominantly fair methodological quality of the included studies, the results suggest that structured training programs may have a positive impact, particularly on physical fitness parameters and kabaddi-specific skills. These findings may assist coaches and kabaddi practitioners in selecting and designing training methods to optimize player performance.

An optimal level of physical fitness is essential for performance in kabaddi due to the high demands it places on strength, endurance, speed, and agility [31]. Among the various physical capacities, strength, speed, and agility were the most frequently assessed, showing improvements in most studies within the experimental groups. Furthermore, between-group comparisons indicated that the different training methods employed were more effective in enhancing these capacities than conventional training or not participating in any training at all. These findings are consistent with results observed in basketball players [32] and tennis players [33], and may be attributed to the implementation of specific training protocols that include functional exercises aimed at improving these capacities, which tend to yield greater benefits than standard routines focused on technical or tactical elements. Moreover, the inclusion of plyometric exercises may have also contributed to the observed improvements, as there is strong evidence supporting the positive effects of this training method, particularly on strength, speed, and agility [34]. On the other hand, flexibility, although only assessed in one study, also showed significant intra- and intergroup improvements favoring the experimental training methods. These differences were more notable in the group that followed the dands and baithaks exercise training, which likely contributed to enhanced flexibility due to the mobility elements integrated into this traditional training modality [20].

The development and mastery of sport-specific technical skills are essential to ensure effective performance across various game situations [35]. Both intra- and intergroup results suggested that all training methods used were more effective than abstaining from any physical activity in improving technical skills. This can be attributed to the fact that even general or non-specific physical training enhances motor coordination, which is a fundamental pillar for the execution of motor skills [36,37]. Furthermore, compared to the group that participated solely in specific kabaddi games, the group that followed a training program incorporating Bulgarian bag exercises integrated with kabaddi-specific drills showed greater improvements in overall game ability. This effect can be explained by the specificity of Bulgarian bag exercises in developing functional strength, grip, core stability, and movement patterns [38,39]. Specifically, in kabaddi, the successful execution of sport-specific skills relies heavily on a combination of technical proficiency and optimal physical conditioning [40].

The available evidence on physiological variables such as VO2_max_ and resting heart rate, as well as hematological parameters including blood cell count, red blood cell count, and hemoglobin levels, suggested significant benefits of Bulgarian Bag Training. Moreover, compared to participants who engaged exclusively in kabaddi-specific training, the positive effects on physical performance were primarily observed in the training method that combined Bulgarian Bag Training with their standard kabaddi-specific routine. These effects may be attributed to the fact that Bulgarian Bag Training incorporates multi-joint functional movements that enhance vital capacity [38], as well as aerobic [41] and anaerobic capacity [42], leading to greater physiological adaptation compared to traditional sport-specific training. In turn, this has been shown to more effectively stimulate the cardiovascular and hematological systems [43]. However, due to the limited number of studies, this review cannot establish a definitive relationship on this matter.

Despite the relevance of this review, several limitations must be acknowledged. First, the included interventions were heterogeneous in terms of type, duration, and intensity, which complicates direct comparisons across studies. Second, the small sample sizes in most studies limit the generalizability of the findings and reduce the statistical power necessary for a meta-analysis. Third, the overall methodological quality of the included studies was fair to low, further restricting the strength of the conclusions. Therefore, future research should focus on conducting high-quality RCTs and exploring alternative training methods to better understand their effects. In addition, future investigations should standardize outcome selection and measurement protocols to improve comparability across studies, and report training dose and progression transparently (e.g., frequency, intensity, time, type, volume, adherence, and adverse events), including follow-up assessments when possible.

## 5. Conclusions

The findings of this systematic review suggest that structured training programs can significantly enhance physical fitness and kabaddi-specific skills among players. Improvements were particularly evident in strength, speed, and agility, as well as in technical performance and certain physiological parameters in several studies. These results indicate that incorporating targeted physical training into routine practice may help optimize athletic performance in kabaddi. Therefore, coaches and physical trainers working with kabaddi players should consider integrating these evidence-based training methods into their conditioning programs. Nevertheless, given the heterogeneity and limited methodological quality of the included studies, further high-quality randomized controlled trials are necessary to validate these findings.

## Figures and Tables

**Table 1:** General characteristics of the included studies.

First Author (Year), Design	Sample	Intervention	Outcomes	Results (*p* < 0.05)
Arjunan (2015) [20] **Design:** RCT	**Participants (*n*):** 45 players (15 EG1; 15 EG2; 15 CON) **Sex:** Male **Age, *years* (range):** 21–28	**Duration:** 6-weeks **Frequency:** 5 days/week **Volume:** NR **Intensity:** NR **EG1:** Rope skipping dands **EG2:** Dands and baithaks exercise training **CON** **Activities:** Do not participate any training	**Physical fitness:** Flexibility Agility	**Intra-group** ↑ Flexibility in *EG1* and *EG2* ↑ Agility in *EG1* and *EG2* **Inter-group:** *EG1* and *EG2* > Agility than *CON* *EG2* > Agility than *EG1* *EG1* and *EG2* > Flexibility than *CON* *EG2* > Flexibility than *EG1*
Nithin et al. (2024) [21] **Design:** RCT	**Participants (*n*):** 30 young adult players (15 EG; 15 CON) **Sex:** Male **Age, *years* (mean; SD):** EG: 20.73 ± 1.83 CON: 20.80 ± 1.69	**Duration:** 12-weeks **Frequency:** 3 days/week **Volume:** 60 min **Intensity:** NR **EG:** Bulgarian Bag training (BBT) **Activities:** following the BBT component, merged with those in the CON to finish the remaining section of their standard kabaddi-specific training routine. **CON** **Activities:** participated only in kabaddi-specific training	**Physical fitness:** Strength: ○ Shoulder Strength ○ Explosive Strength ○ Muscular Strength Agility **Physiological Variables:** Resting Heart Rate VO2_Max_ **Hematological Variables:** White Blood Cells Red Blood Cells Hemoglobin **Sports Performance:** Playing Ability	**Intra-group** ↑ Shoulder Strength in *EG* ↑ Explosive Strength in *EG* ↑ Muscular Strength in *EG* ↑ Agility in *EG* ↑ Resting Heart Rate in *EG* ↑ VO2_Max_ in *EG* ↑ White Blood Cells in *EG* ↑ Red Blood Cells in *EG* ↑ Hemoglobin in *EG* ↑ Playing Ability in *EG* **Inter-group:** *EG* > Agility than *CON* *EG* > Shoulder, Explosive and Muscular Strength than *CON* *EG* < Resting Heart Rate than *CON* *EG* > VO2_Max_ than *CON* *EG* > White Blood Cells, Red Blood Cells and Hemoglobin than *CON* *EG* > Playing Ability than *CON*
Irandoust & Taheri (2014) [22] **Design:** RCT	**Participants (*n*):** 48 elite players (12 EG1; 12 EG2; 12 EG3; 12 CON) **Sex:** Male **Age, *years* (mean; SD):** 23.58 ± 0.51	**Duration:** 8-weeks **Frequency:** 3 days/week **EG1:** Strength training **Volume:** NR **Intensity:** 60–86% 1 RM **EG2:** Plyometric training **Volume:** NR **Intensity:** low-high exercises **EG3:** Combined (strength + plyometric) training **Volume:** NR **Intensity:** 60–86% 1 RM; low-high exercises **CON** **Activities:** Do not participate any training	**Physical fitness:** Strength: ○ Leg explosive power Speed Agility	**Intra-group** ↑ Leg explosive power in *EG1*, *EG2* and *EG3* ↑ Agility in *EG1*, *EG2* and *EG3* ↑ Speed in *EG1*, *EG2* and *EG3* **Inter-group:** *EG1*, *EG2* and *EG3* > leg explosive power than *CON* *EG1*, *EG2* and *EG3* > Speed than *CON* *EG1*, *EG2* and *EG3* > Agility than *CON*
Karuppaiah & Kumar (2022) [23] **Design:** RCT	**Participants (*n*):** 30 college level players (15 EG; 15 CON) **Sex:** Male **Age, *years* (range):** 18–25	**Duration:** 12-weeks **Frequency:** NR **Volume:** NR **Intensity:** NR **EG:** Circuit training **CON** **Activities:** Do not participate any training	**Sports Performance:** Knee Hold Kicking	**Intra-group** ↑ Knee Hold in *EG* ↑ Kicking in *EG* **Inter-group:** NR
Shanmugam et al. (2024) [24] **Design:** RCT	**Participants (*n*):** 80 college level players (20 EG1; 20 EG2; 20 EG3; 20 CON) **Sex:** Male **Age, *years* (range):** 18–28	**Duration:** 12-weeks **Frequency:** NR **Volume:** 60 min **Intensity:** NR **EG1:** Game specific with unilateral training **EG2:** Game specific with bilateral training group **EG3:** Game specific with complex training group **CON** **Activities:** not inducted into any specific training program	**Physical fitness:** Speed Aerobic capacity	**Intra-group:** ↑ Speed *EG1*, *EG2* and *EG3* ↑ Aerobic capacity *EG1*, *EG2* and *EG3* **Inter-group:** NR
Sureshkumar et al. (2022) [25] **Design:** RCT	**Participants (*n*):** 60 college level players (15 EG1; 15 EG2; 15 EG3; 15 CON) **Sex:** Women **Age, *years* (range):** 18–25	**Duration:** 8-weeks **Frequency:** 3 days/week **Volume:** 60 min **Intensity:** NR **EG1:** Interval training **EG2:** Continues training **EG3:** Combined interval and continuous training **CON** **Activities:** do not participate any kind of activities.	**Physical fitness:** Stride Length	**Intra-group** NR **Inter-group:** *EG1*, *EG2* and *EG3* > Stride Length than *CON*
Sureshkumar et al. (2023) [26] **Design:** RCT	**Participants (*n*):** 45 college level players (15 EG1; 15 EG2; 15 CON) **Sex:** Women **Age, *years* (range):** 18–25	**Duration:** 12-weeks **Frequency:** 3 days/week **Volume:** 60 min **Intensity:** NR **EG1:** Interval training **EG2:** Continues training **CON** **Activities:** do not participate any kind of activities.	**Physical fitness:** Strength: ○ Leg explosive power Agility Speed **Sports Performance:** Toe touch Hand touch Ankle touch	**Intra-group** NR **Inter-group:** *EG1* and *EG2* > Agility than *CON* *EG1* and *EG2* > Leg explosive power than *CON* *EG1* and *EG2* > Speed than *CON* *EG1* and *EG2* > Hand touch than *CON* *EG1* and *EG2* > Toe touch *CON* *EG1* and *EG2* > Ankle touch than *CON*
Tandel (2018) [27] **Design:** RCT	**Participants (*n*):** 30 young adult players (15 EG; 15 CON) **Sex:** Male **Age, *years* (range):** 17–21	**Duration:** 6-weeks **Frequency:** 3 days/week **Volume:** 60–75 min **Intensity:** NR **EG:** Strength training **CON** **Activities:** Do not participate any training	**Physical fitness:** Strength: ○ Muscular Endurance Agility Speed	**Intra-group** ↑ Muscular Endurance in *EG* ↑ Agility in *EG* ↑ Speed in *EG* **Inter-group:** *EG* > Agility than *CON* *EG* > Muscular Endurance than *CON* *EG* > Speed than *CON*
Thinakaran et al. (2024) [28] **Design:** RCT	**Participants (*n*):** 45 school level kabaddi players (15 EG1; 15 EG2; 15 CON) **Sex:** Male **Age, *years* (range):** 14–17	**Duration:** 12-weeks **Frequency:** 5 days/week **Volume:** 80 min **Intensity:** NR **EG1:** Tabata training **Activities:** The load was increased by every four weeks of their training program **EG2:** Resistance training **Activities:** The load was increased by every four weeks of their training program **CON** **Activities:** participated only in kabaddi-specific training	**Physical fitness:** Speed	**Intra-group:** ↑ Speed *EG1* and *EG2* **Inter-group:** *EG1* and *EG2* > Speed than *CON*
Upadhyay (2023) [29] **Design:** RCT	**Participants (*n*):** 45 college level players (15 EG1; EG2; 15 CON) **Sex:** Male **Age, *years* (range):** 19–23	**Duration:** 6-weeks **Frequency:** 4 days/week **Volume:** NR **Intensity:** NR **EG1:** Speed agility quickness training **EG2:** Plyometric training **CON** **Activities:** asked to refrain from any special training except their leisure time pursuit as college students.	**Physical fitness:** Strength: ○ Power Speed Agility	**Intra-group** ↑ Speed *EG1* and *EG2* ↑ Agility *EG1* and *EG2* ↑ Power *EG1* and *EG2* **Inter-group:** *EG1* and *EG2* > speed than *CON* *EG1* and *EG2* > agility than *CON* *EG1* and *EG2* > power than *CON*

>: Greater; <: Lower; ↑: Increment; CON: Control Group; EG: Experimental Group; NR: Not Reported; RCT: Randomized controlled trial.

**Table 2:** Quality Assessment of Randomized Controlled Trials.

Authors (Year)	Items												Quality Rating
	1	2	3	4	5	6	7	8	9	10	11	Score	
Arjunan (2015) [20]	Y	+	−	−	−	−	−	+	+	+	−	4/10	Fair
Nithin et al. (2024) [21]	Y	+	+	+	−	−	−	+	+	+	+	7/10	Good
Irandoust & Taheri (2014) [22]	Y	+	−	−	−	−	−	+	+	+	+	5/10	Fair
Karuppaiah & Kumar (2022) [23]	Y	+	−	−	−	−	−	+	+	−	−	3/10	Fair
Shanmugam et al. (2024) [24]	Y	+	−	−	−	−	−	+	+	−	+	4/10	Fair
Sureshkumar et al. (2022) [25]	Y	+	−	−	−	−	−	+	+	+	−	4/10	Fair
Sureshkumar et al. (2023) [26]	Y	+	−	−	−	−	−	+	+	+	−	4/10	Fair
Tandel (2018) [27]	Y	+	−	−	−	−	−	+	+	+	−	4/10	Fair
Thinakaran et al. (2024) [28]	Y	+	−	−	−	−	−	+	+	+	−	4/10	Fair
Upadhyay (2023) [29]	Y	+	−	−	−	−	−	+	+	+	−	4/10	Fair

**Note.** Eligibility criteria item does not contribute to total score. Items: (1) eligibility criteria; (2) randomization; (3) concealed allocation; (4) similarity at baseline; (5) subjects blinding; (6) therapists blinding; (7) assessors blinding; (8) one key outcome measured in >85% of subjects; (9) intention-to-treat analysis; (10) between-group statistical results for one key outcome; (11) measures of variability and point measures for one key outcome. “+” indicates that the criterion was met; “−” indicates that the criterion was not met.

## Data Availability

The data that support the findings of this study are available from the corresponding author (D.G.-D.), upon reasonable request.

## Supplementary Materials

The following supporting information can be downloaded at: https://www.mdpi.com/article/10.3390/sports14010037/s1, File S1: The PRISMA 2020 statement: an updated guideline for reporting systematic reviews.
